# Transatmospheric ileal stoma manometry can be applied for the early detection of stoma outlet obstruction

**DOI:** 10.3389/fonc.2023.1187858

**Published:** 2023-07-31

**Authors:** Xiaowei Wang, Yizhi Wang, Beibei Lin, Yue Liu, Jin Gu, Limian Ling, Dong Xu, Kefeng Ding

**Affiliations:** ^1^ Department of Anorectal Surgery, The First People’s Hospital of Wenling, Wenling, Zhejiang, China; ^2^ Department of Hepatobiliary and Pancreatic Surgery, The Second Affiliated Hospital, Zhejiang University School of Medicine, Hangzhou, Zhejiang, China; ^3^ Department of Colorectal Surgery and Oncology Key Laboratory of Cancer Prevention and Intervention, Ministry of Education, The Second Affiliated Hospital, Zhejiang University School of Medicine, Hangzhou, Zhejiang, China

**Keywords:** stoma outlet obstruction, manometry, ileostomy, rectal cancer, eras

## Abstract

**Background:**

Stoma outlet obstruction (SOO) is a common complication of diverting ileostomy and usually detected at the advanced stage when the intestine is obviously obstructed. The objective of this study is to explore the efficacy of transatmospheric ileal stoma manometry (TISM) in early detection of SOO before the manifestation of intestinal obstruction.

**Methods:**

A single-center prospective study was performed in patients scheduled to undergo reversal ileostomy and laparoscopic anterior rectal resection and diverting ileostomy in Second Affiliated Hospital of Zhejiang University School of Medicine from 1^st^ July 2022 to 31^st^ December 2022. The stoma pressure was measured by TISM at different time points.

**Results:**

The mean stoma pressure of the 30 patients before reversal ileostomy was 5.21 cmH_2_O which was considered as normal standard of stoma pressure, and ranged from 1.2 to 8.56 cmH_2_O. After excluding two patients with anastomotic leakage, a total of 38 patients who were subjected to laparoscopic anterior rectal resection and diverting ileostomy were further included in this study. The incidence of anastomotic leakage was 5% and that of SOO was 12.5%. The mean postoperative obstruction time was 5.2 (3-7) days and the mean time from elevated stoma pressure to diagnosed as SOO was 2.8 (2-4) days in the five patients who developed SOO. The pressure measured at the third stoma manometry time point (second day after return of gut function) (10.23 vs. 6.04 cmH_2_O, *p*<0.001) and the postoperative hospital stay (10 vs. 8.49 days, *p*=0.028) showed significantly difference between the SOO and non-SOO groups. The pressures measured at the first time point (before return of gut function) (4 vs. 4.49 cmH_2_O, *p*=0.585), the second time point (the day of return of gut function) (6.8 vs. 5.62 cmH_2_O, *p*=0.123), and the fourth time point (discharge day) (5.88 vs. 5.9 cmH_2_O, *p*=0.933) showed no significant difference in both groups.

**Conclusion:**

TISM can be utilized for early detection of SOO and can be incorporated as a novel diagnostic method together with abdominal CT scan to realize the goal of ERAS.

## Introduction

The incidence of the overall anastomotic leakage after low anterior resection for rectal cancer is 10-20% (1–3). Transanal drainage tube may be ineffective at times against anastomotic leakage and diverting ileostomy is recommended for low anterior resection of rectal cancer to prevent postoperative anastomotic leakage (4–6). However, stoma outlet obstruction (SOO) is a common complication of diverting ileostomy with an incidence rate from 5.6% to 18.4%, and usually develops within 2 weeks after operation (7–9). Furthermore, SOO is usually diagnosed when patients present typical symptoms of obstruction, such as abdominal pain and distention, nausea and vomiting, and can be relieved by expectant treatment such as insertion of an anal catheter to the proximal small intestine and fecal fluid will be immediately drawn out to relieve obstruction (10). SOO can cause a huge economic burden for patients due to the increased treatment costs and longer hospital stays, thus early detection of SOO can be necessary. Although daily postoperative abdominal CT scan can reliably detect obstruction at early stage, it is not a feasible option due to tedious process and large doses of radiation. Moreover, proximal small bowel manometry has been previously used as an effective method to diagnose small bowel obstruction, and there have been studies of aberrant ileal manometry findings in irritable bowel syndrome and chronic idiopathic constipation (11–13). In this study, we aimed to explore the feasibility of using ileal stoma manometry for early detection of stoma obstruction after ileostomy.

## Patients and methods

### Patients

*Cohort 1*: 30 patients scheduled to undergo reversal ileostomy after low anterior resection for rectal cancer were recruited from July 1^st^ 2022 to December 31^st^ 2022. The first radical surgery was either laparoscopic or robotic. The exclusion criteria were as follows (1): incomplete obstruction of the stoma which can be confirmed *via* enhanced abdominal CT scan with no sign of complete intestinal obstruction (2), preoperative albumin below 30 g/l, and (3) local stoma or systemic infection.

*Cohort 2*: 40 patients with rectal cancer who underwent laparoscopic anterior rectal resection and diverting ileostomy from July 1^st^ 2022 to 31^st^ December 2022 were also enrolled. The exclusion criteria were as follows (1): open surgery or conversion to open surgery (2), non-neoplastic diseases (3), postoperative anastomotic leakage, massive bleeding, or death.

### Settings and groups

The single-center prospective study was performed at Second Affiliated Hospital of Zhejiang University School of Medicine. The ileal stoma pressure was measured by TISM and the main outcome measure was the stoma pressure at different time measurement points. Patients were divided into the SOO and non-SOO groups as appropriate.

### Diagnostic criteria of outlet obstruction

(1) Typical clinical symptoms, such as abdominal pain, nausea and vomiting, abdominal distension (2), abdominal CT scan showing small bowel obstruction at the stoma.

### Methods of TISM

**Figure 1:**
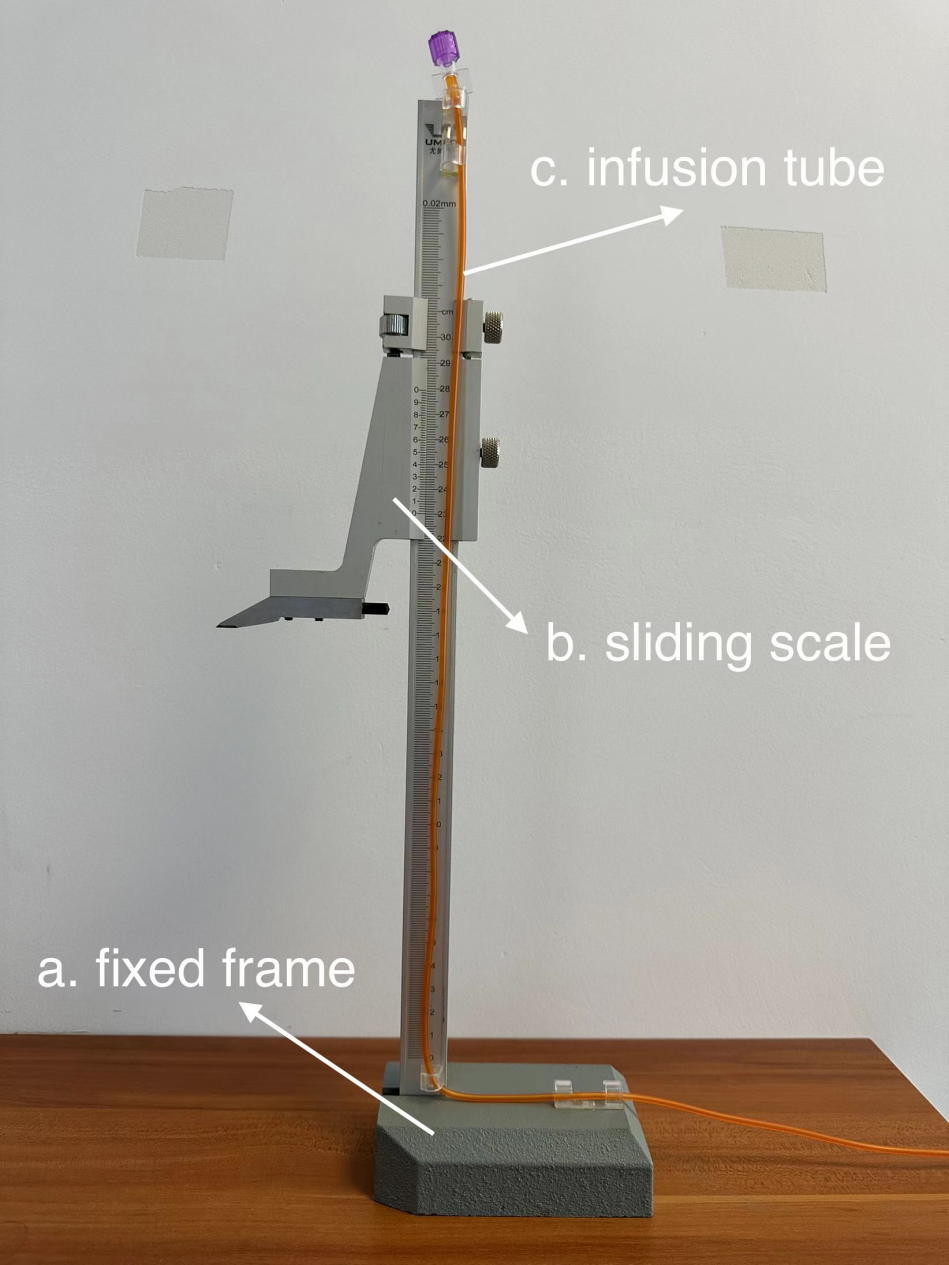
This is the device which is modified for TISM. **(A)** fixed frame. **(B)** sliding scale. **(C)** infusion tube.

The manometry device consists of a fixed frame, a sliding scale (accuracy 0.02 mm), and an infusion tube (diameter 2 mm, length 80 cm) to measure the pressure of the proximal intestines at the stoma relative to the standard atmospheric pressure (**Figure 1**).

The patient was placed in supine position without any pillow, and the stoma bag was removed to expose and then clean the stoma. The frame was fixed on the right side of the patient, and the height of the skin at the lower edge of the stoma was measured. The thickness of the abdominal wall was estimated according to the preoperative abdominal CT, and the infusion tube was gently inserted 4-8 cm into the small intestine at the proximal end of the stoma. After injecting 1.5 ml saline into the intestinal cavity, the water column was seen to slide down to a fixed position. The difference between the height of the water column and the height of the skin at the lower edge of the stoma was calculated as the pressure of the intestinal cavity at the particular time point.

### Time points of measuring stoma pressure

Stoma manometry was performed once or twice preoperatively, and at the following time points after the operation (1): before return of gut function (2), on the day of return of gut function (3), second day after return of gut function, and (4) the day of discharge. When the stoma pressure was greater than 8.56 cmH_2_O, it was measured 2-4 times more, including before and after the treatment for SOO. The pressure was measured three times at each time point to achieve the average value. However, in our actual operation, the second and third measurements yielded lower values compared to the first. Considering the possible effect of the stoma on manometry reading, the first measurement was used for the analysis.

### Statistical analysis

The data were expressed as mean ± standard deviation (SD). For univariate analysis, Student’s t test and Mann-Whitney U test was used to compare the two groups. The optimal cutoff values to predict SOO were calculated with the maximum sum of sensitivity and specificity using the Youden index. *P* value<0.05 was considered statistically significant. All statistical analyses were performed using SPSS 26.0 (IBM Corporation, Armonk, NY, USA).

## Results

### Clinical characteristics of the patients

30 patients scheduled to undergo reversal ileostomy after low anterior resection for rectal cancer were selected. The mean age of the patients at diagnosis was 64.53 (39–89) years and the mean time to stoma closure was 118.4 (55–338) days after initial-operation. In addition, all stomas were located in the right lower abdomen. In order to exclude the influence of other relevant factors, we first measured the pressure range in the small intestine proximal to the stoma. As shown in **Table 1**, the mean stoma pressure of the patients before reversal ileostomy was 5.21 cmH_2_O ranged from 1.2-8.56 cmH_2_O. This value will be used as the reference for our subsequent study. The average time spent on pressure measurement was 296.2 (195–506) s. Other pre-operative clinical characteristics of the included patients were also summarized in **Table 1**.

**Table 1:** Clinical characteristics of the patients before reversal ileostomy.

Characteristics	Range
**Age (years)**	64.53 (39–89)
**Gender (male/female)**	18/12
**Stomal site (right lower/others)**	30/0
**Stoma closure time (day)**	118.40 (55–338)
**Stoma pressure value before reversal ileostomy (cmH_2_O)**	5.21 (1.2-8.56)
**Approach of radical surgery (laparoscopy or robot/open)**	30/0
**Preoperative WBC (10E^9^/L)**	5.58 (4.1-11.5)
**Preoperative ALB (g/l)**	37.92 (31.9-45.8)
**Time spent on pressure measurement (seconds)**	296.2 (195–506)

After excluding two patients with anastomotic leakage, 38 patients who were subjected to laparoscopic anterior rectal resection and diverting ileostomy were included in the subsequent analysis. The incidence of anastomotic leakage was 5% and that of SOO was 12.5%. The mean postoperative obstruction time was 5.2 (3–7) days and the mean time from elevated stoma pressure to diagnosis of SOO was 2.8 (2–4) days in the five SOO patients. The mean age of the 38 patients was 67.89 (48–90) years and the mean BMI was 23.08 (17.92-28.33) kg/m^2^. All stomas were located in the right lower abdomen. The mean time to deflation was 1.32 (1–2) days, and the mean postoperative hospital stay was 8.68 (5–23) days, with no unplanned secondary surgeries. Clinical and surgical characteristics of the two groups of patients are shown in **Table 2**.

**Table 2:** Clinical and surgical characteristics of SOO and non-SOO groups.

Characteristics	Range
**Age (years)**	67.89 (48–90)
**Gender (male/female)**	29/9
**Body mass index (kg/m^2^)**	23.08 (17.92-28.33)
**Diabetes (yes/no)**	7/31
**Hypertension (yes/no)**	15/23
**ASA (grade 1-2/3-4)**	36/2
**Preoperative chemoradiotherapy (yes/no)**	4/34
**Operative time (min)**	223.16 (149–365)
**Blood loss rate (%)**	8.29 [(-11.61)-22.14]
**Anastomosis (double stapling technique/hand-sewn)**	38/0
**Surgical approach (robot/laparoscopy)**	8/30
**Stoma outlet obstruction (yes/no)**	5/33
**Time of return of gut function (day)**	1.32 (1–2)
**Stomal site (right lower/others)**	38/0
**Meal times (day)**	3 (2–7)
**Postoperative hospital stay duration (day)**	8.68 (5–23)
**Stage (I/II/III/IV)**	16/15/7/0
**Incidence of reoperation**	0

### Univariate analysis of stoma pressure

**Figure 2:**
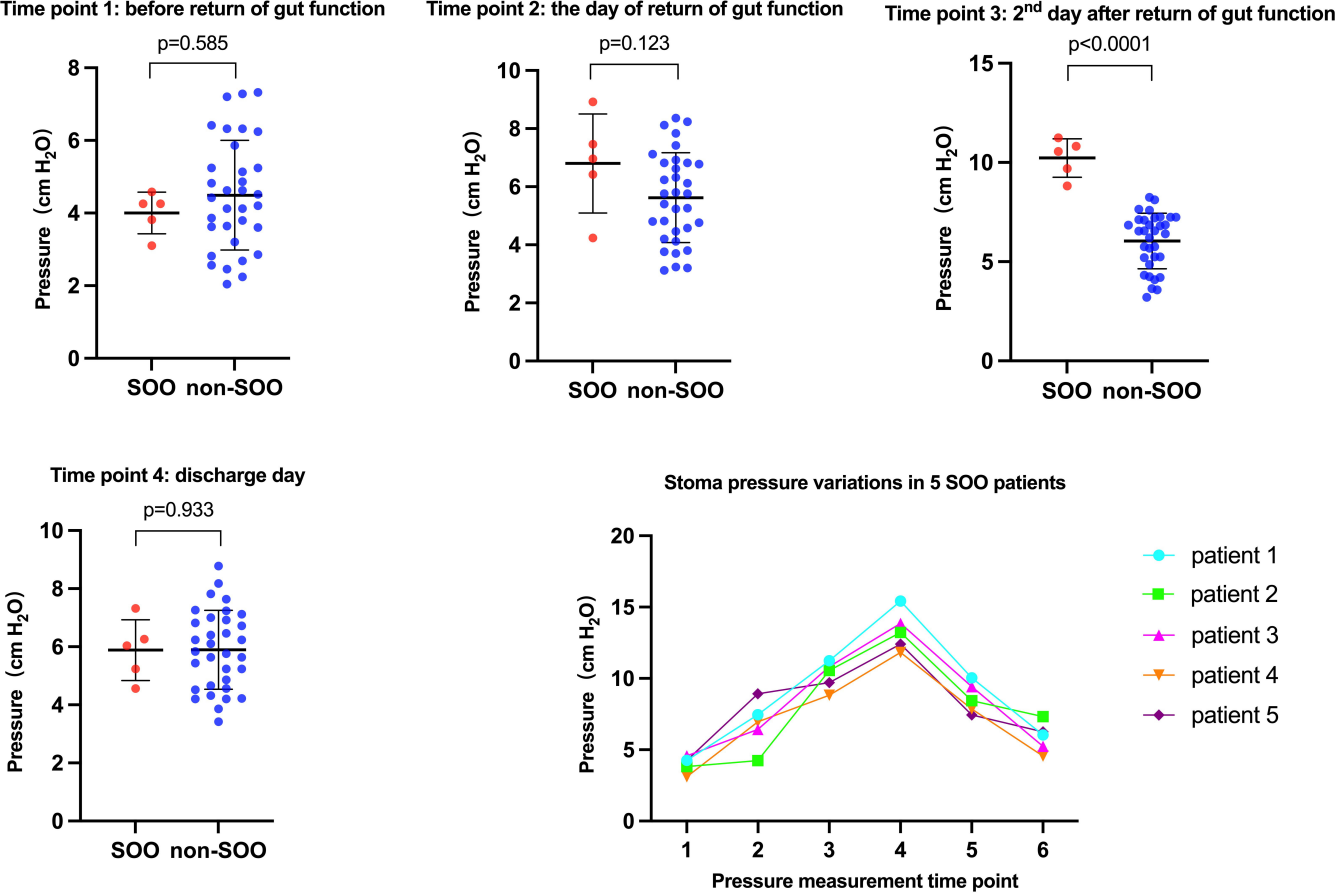
These are the figures that univariate analysis between the SOO group and non-SOO group at different stoma pressure measurement time points and the variations in stoma pressure in the five SOO patients.

The patients with SOO had significantly higher stoma pressure on the second day after return of gut function compared to non-SOO group (10.23 vs. 6.04 cmH_2_O, *p*<0.001). In contrast, the stoma pressure before return of gut function (4 vs. 4.49 cmH_2_O, *p*=0.585), the day of return of gut function (6.8 vs. 5.62 cmH_2_O, *p*=0.123), and the day of discharge (5.88 vs. 5.90 cmH_2_O, *p*=0.933) did not show significant difference between the two groups (**Figure 2**, **Table 3**).

**Table 3:** Univariate analysis for stoma pressure of SOO group and non-SOO group.

Time points of stoma pressure measurement	Range of SOO group	Range of non-SOO group	*p*-value
**Time point 1 (before return of gut function) pressure (cmH_2_O)**	4.00 (3.1-4.58)	4.49 (2.04-7.32)	0.585
**Time point 2 (the day of return of gut function) pressure (cmH_2_O)**	6.8 (4.24-8.92)	5.62 (3.12-8.36)	0.123
**Time point 3 (2^nd^ day after return of gut function) pressure (cmH_2_O)**	10.23 (8.82-11.24)	6.04 (3.2-8.24)	**0.000**
**Time point 4 (discharge day) pressure (cmH_2_O)**	5.88 (4.56-7.32)	5.90 (3.42-8.78)	0.933
**Maximum stoma pressure during hospitalization (cmH_2_O)**	13.34 (11.82-15.42)	7.04 (4.86-8.78)	**0.000**

The bold values mean that p values were less than 0.05 and were statistically significant.

The patients in the SOO group did not develop obstruction before and on the day of return of gut function. On the second day after return of gut function, some patients gradually began to develop stoma obstruction, although none exhibited any symptoms of intestinal obstruction. The manometry readings were taken daily until the patients showed symptoms of obstruction, and abdominal CT scan indicated that the obstruction site was in the stoma. Since the stoma pressure had peaked at this point, the patients were treated by inserting an anal tube. Then the anal tube was removed once the patient’s obstructive symptoms were relieved, and the pressure was measured daily until the patient was discharged. The optimal cutoff pressure value to predict SOO was 8.53cmH_2_O. The variations in stoma pressure in the five SOO patients were presented in **Figure 2**.

### Univariate analysis of other clinical factors

Patients with SOO had significantly longer mean hospital stay compared to those without SOO (10 vs. 8.49 days, *p*=0.028). However, gender (*p*=0.837), operation time (205.8 vs. 225.79 min, *p*=0.475), intraoperative blood loss rate (7.78 vs. 8.37%, *p*=0.645), time to return of gut function (1.4 vs. 1.3 days, *p*=0.738) and highest postoperative WBC (9.36 vs. 10.69 10E9/L, *p*=0.331) were not significantly different between the two groups (**Table 4**).

**Table 4:** Univariate analysis of variables between SOO group and non-SOO group.

Characteristics	SOO group	non-SOO group	*p*-value
**Age (years)**	62.60	68.70	0.271
**BMI (kg/m^2^)**	23.50	23.01	0.501
**Gender (male/female)**	4/1	25/8	0.837
**Preoperative chemoradiotherapy (yes/no)**	0/5	4/29	0.417
**Operation time (min)**	205.80	225.79	0.475
**Blood loss rate (%)**	7.78	8.37	0.645
**Surgical approach (robotic/laparoscopy)**	0/5	8/25	0.221
**Time of postoperative return of gut function (day)**	1.40	1.30	0.738
**Meal times (day)**	3.00	3.00	0.675
**Postoperative hospital stay duration (days)**	10.00	8.49	0.028
**Preoperative WBC (10E^9^/L)**	5.58	7.12	0.252
**Highest postoperative WBC (10E^9^/L)**	9.36	10.69	0.331
**Highest postoperative CRP (mg/l)**	67.00	79.70	0.557
**Preoperative Alb (g/l)**	37.92	39.26	0.31
**Minimum postoperative Alb (g/l)**	33.30	31.88	0.235
**Alb descending rate (%)**	12.17	18.31	0.084

## Discussion

Although the exact pathological reason causing SOO remains indistinct, previous studies have showed that contraction of the rectus abdominis muscle and infection might be the potential reasons (9, 14, 15). Recently, a retrospective study (unpublished study) conducted by our team included 306 patients who underwent laparoscopic anterior rectal resection and diverting ileostomy from Aug 2019 to Aug 2022 and 28 of them developed SOO. Patients were divided into the SOO and non-SOO groups. We found that no significantly differences were exhibited in the maximum abdominal wall thickness of stoma, the width of the abdominal wall defect and small bowel torsion by abdominal CT scan between the two groups. Moreover, our unpublished study also indicated that rectus abdominis muscle contraction may not play a significant role in the development of SOO which was opposite to the previous study. However, our study found that stoma edema is exactly a direct cause of stoma obstruction which may be caused by too much bowel and mesentery is dragged out during ileostomy and subsequent infection.

**Figure 3:**
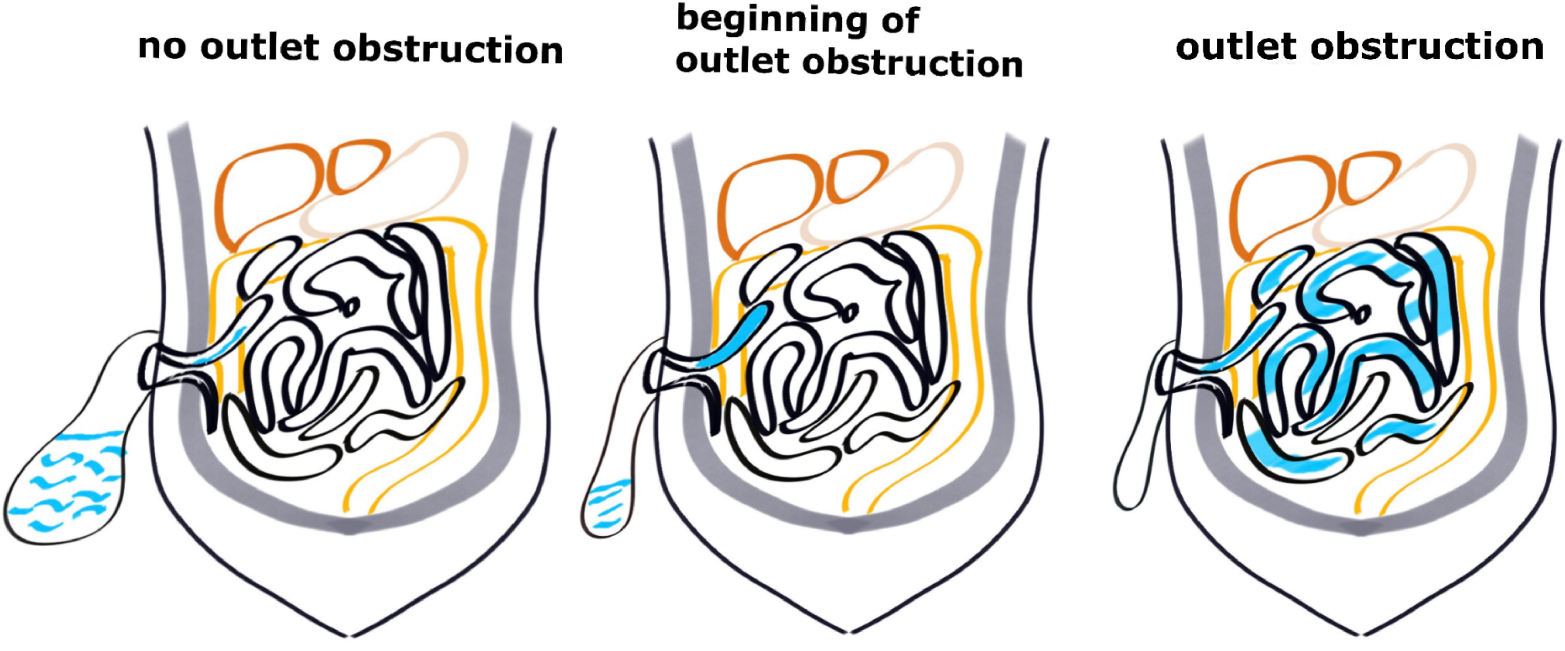
Diagram of the different stages of outlet obstruction.

In patients without SOO, when the proximal intestinal canal accumulates fecal water and the intestinal lumen pressure reaches a critical value, the stoma opens and the fecal water flows into the stoma bag and then intestinal lumen pressure decreases, and the process is repeated. In contrast, in patients with SOO, due to stoma edema or small stoma opening, fecal water cannot be discharged, and the pressure in the proximal intestinal canal continues to rise, then the obstruction gradually spreads to the proximal small intestine, and patients gradually develop symptoms of intestinal obstruction such as abdominal distension and vomiting (**Figure 3**). In our present study, the patients with SOO had significantly higher stoma pressure on the second day after return of gut function compared to those without SOO. Because the five patients already had SOO on the second day after return of gut function. The fecal water gradually accumulated in the proximal small intestine at the stoma and could not be discharged, the intestinal lumen pressure increased, and the intestinal lumen gradually expanded, at which time the measured pressure was significantly greater than the upper limit in cohort 1 (8.56 cmH_2_O). Then the obstruction gradually progressed to the proximal small intestine, and finally symptoms of intestinal obstruction such as abdominal distention and vomiting appeared after 2-4 days. In patients without SOO, the intestinal lumen was expanded to a certain extent and fecal water discharged through the stoma into the stoma bag, so that the intestinal lumen pressure was always maintained at a normal range (1.2-8.56 cmH_2_O).

Based on our findings, we recommended intestinal pressure measurement at the stoma more than 8.53 cmH_2_O, and dilatation of the proximal small intestine at the stoma in abdominal CT scan or ultrasound as the novel diagnostic criteria for SOO. If SOO is detected at an early stage, typical symptoms of intestinal obstruction such as nausea and vomiting may not be considered as necessary factors for diagnosis.

Effective early intervention following detection of elevated stoma pressure may halt further obstruction and accelerate patient recovery before the symptoms of intestinal obstruction. Patients with obstruction usually develop edema and inflammation in the stoma intestine, which can be relieved by corticosteroids and the use of an anal tube (16). Studies show that moderate doses of corticosteroids during major surgery in short courses can reduce postoperative infection without any significant risk of anastomotic leakage or bleeding, although this has not been validated in a large randomized controlled trial study (17). A defunctioning stoma did not affect the anastomotic leakage risk, it significantly reduced its severity (6, 18) .Therefore, the use of corticosteroids still has to take into account the risk of anastomotic leakage and needs to be used with caution.

Traditional manometry requires placing a catheter into the target site, inserting a ventilation tube, and then filling and deflating the catheter, which may cause discomfort to the patient and prolong the time to measure (19). The technical difficulties, prolonged duration and high costs of stoma manometry limit its clinical application. Daily review of abdominal CT scan is able to detect stoma dilatation before the patient develops symptoms of obstruction, enabling timely diagnosis of stoma obstruction in the early stages of obstruction. However, this can lead to a waste of medical resources and increase the cost of patient care. Compared to the above two methods of examinations, TISM has the advantages of portability (bedside pressure measurement), short manometry time (average time spent on pressure measurement was 296.2 (195–506) s), high accuracy, low cost and low stimulation to the stoma. The most common technical issue with manometry is that the end of the infusion tube fails to enter the intestinal cavity through the rectus abdominis muscle since it is coiled at the abdominal wall of the intestinal cavity. Therefore, we inserted the infusion tube and then probed the intestinal cavity with the little finger, and nudged the infusion tube into the cavity if it was coiled. In fact, almost 30% of the patients needed finger-assisted insertion of the infusion tube into the intestinal cavity. Therefore, we subsequently used a guidewire to assist tube insertion in order to avoid this situation. The pressure measurement catheter is a hollow tube, when the stoma is measured, there will inevitably be some gas or liquid escape, which is why the pressure measured in the second and third measurement is lower than the first one, even the reduction is small, but the first measurement is still relatively accurate. In the current study, the rate of SOO was 12.5%, between 5.6% and 18.4% as previously studies reported, with no clear evidence that TISM artificially increases the probability of obstruction.

We propose to include TISM as a supplement to enhanced recovery after surgery (ERAS) in clinical practice. Also, TISM can be used as an effective part of multimodal prehabilitation to improve functional capacity and reduce postoperative complication rates of patients who receive stroma reduction surgery. A recent meta-analysis included three randomised controlled trials indicated the potential advantages of multimodal prehabilitation in clinical application of colorectal surgery (20). In this study, the mean time from elevated stoma pressure to diagnosis of SOO was 2.8 (2–4) days. When patients were diagnosed with SOO, it took 3-5 days for the symptoms of intestinal obstruction to disappear after active treatment. Once SOO occurred, it delayed the recovery of patients and prolonged their hospital stay, thus violating the goal of ERAS. In clinical practice, for patients with rectal cancer who undergo laparoscopic anterior rectal resection and diverting ileostomy, daily stoma manometry can be conducted at the bedside postoperatively. When the measured pressure is greater than 8.53 cmH_2_O and the following abdominal CT indicates dilatation of the intestinal lumen at the stoma, the patient can be diagnosed with early SOO without presenting typical signs of intestinal obstruction. Then we should take early intervention measures such as insert an anal tube for treatment, therefore avoiding unnecessary fasting and parenteral nutrition which central venus catheterization may be needed, and remove the anal tube after 3-5 days. In the present study, for example, one patient with elevated stoma pressure and dilatation of the stoma lumen suggested by abdominal CT, we timely treated the patient with early insertion of an anal tube without fasting or parenteral nutrition, and the abdominal CT review before discharge did not show intestinal obstruction, and the patient discharged 7 days after surgery without any trouble. The length of hospital stay was significantly shorter compared to other 5 patients who were treated only after presenting the symptoms of small bowel obstruction.

For the time to come, we will conduct a large cohort and multicenter prospective study to measure the pressure using TISM in patients with rectal cancer who underwent laparoscopic anterior rectal resection and diverting ileostomy. When we find that the stoma pressure relatively elevates and the examination (CT or ultrasound) suggests stoma dilatation, we immediately give symptomatic treatment such as inserting an anal tube, decongesting, avoiding fasting and parenteral nutrition, and removing the anal tube after the edema at the stoma has subsided. Based on the results of this study, our subsequent study will investigate whether early interventional treatment can reduce the rate of stoma outlet obstruction, accelerate patient early recovery, and reduce patient hospitalization time and costs.

This study still has some limitations. The stoma of patients undergoing open surgery for manometry has not been included in this study. However, based on the limited comparison of stoma pressure after open (8 patients) and laparoscopic surgery (our unpublished study), we do not believe there is a significant difference between the two groups. Meanwhile, the stoma of the colon has not been pressure measured in our present study. Future multicenter randomized clinical trials of large sample size may be needed to achieve a more precise and universal conclusion.

## Conclusions

TISM can be used as a supplementary method for the early detection of SOO and allow timely treatment of the patients before they develop symptoms of obstruction.

## Data availability statement

The original contributions presented in the study are included in the article/supplementary material. Further inquiries can be directed to the corresponding authors.

## Ethics statement

The studies involving human participants were reviewed and approved by Second Affiliated Hospital of Zhejiang University. The patients/participants provided their written informed consent to participate in this study.

## Author contributions

XW wrote the original paper, analyzed data and completed the tables and figures, XW and YW collected data. KD and DX designed research, performed research, analyzed and interpreted data, critically reviewed the manuscript. All authors contributed to the article and approved the submitted version.
